# Application of 2,3-Naphthalenediamine in Labeling Natural Carbohydrates for Capillary Electrophoresis 

**DOI:** 10.3390/molecules17067387

**Published:** 2012-06-15

**Authors:** Chien-Yuan Kuo, Shwu-Huey Wang, Chunchi Lin, Sylvain Kuo-Shiang Liao, Wei-Ting Hung, Jim-Min Fang, Wen-Bin Yang

**Affiliations:** 1 The Genomics Research Center, Academia Sinica, 128, Academia Road, Section 2, Taipei 115, Taiwan; 2 Core Facility Center, Office of Research and Development, Taipei Medical University, 250, Wu-Hsing Street, Taipei 110, Taiwan; 3 Department of Chemistry, National Taiwan University, No. 1, Roosevelt Road, Section 4, Taipei 106, Taiwan

**Keywords:** capillary electrophoresis, 2,3-naphthalenediamine, medicinal saccharides, enantioseparation, glucanases

## Abstract

Neutral and acidic monosaccharide components in *Ganoderma lucidum* polysaccharide are readily labeled with 2,3-naphthalenediamine, and the resulting saccharide-naphthimidazole (NAIM) derivatives are quantified by capillary electrophoresis (CE) in borate buffer. Using sulfated-α-cyclodextrin as the chiral selector, enantiomers of monosaccharide-NAIMs are resolved on CE in phosphate buffer, allowing a simultaneous determination of the absolute configuration and sugar composition in the mucilage polysaccharide of a medicinal herb *Dendrobium* *huoshanense*. Together with the specific enzymatic reactions of various glycoside hydrolases on the NAIM derivatives of glycans, the structures of natural glycans can be deduced from the digestion products identified by CE analysis. Though heparin dissachrides could be successfully derived with the NAIM-labeling method, the heparin derivatives with the same degree of sulfation could not be separated by CE.

## 1. Introduction

It is important to determine the composition and configuration of carbohydrates because carbohydrates are one of the most important components in organisms. Capillary electrophoresis (CE) is a powerful tool for the analysis of biomolecules; however, most oligo- and polysaccharides have problems in CE detection due to lack of chromophore or charged moiety. To aid detection of saccharides in CE, a large number of charged, UV active and fluorescent reagents, such as 2-amino-pyridine (2-AP), 2-aminobenzoic acid (2-AA), 2-aminobenzamide (2-AB), 9-aminoacridone (AMAC), 7-aminonaphthalene-1,3-disulfonic acid (ANDSA), rhodamine 110 (Rho 110), 8-aminonaphthalene-1,3,6-trisulfonate (ANTS), 8-aminopyrene-1,3,6-trisulfonate (APTS), and 1-phenyl-3-methyl-2-pyrazolin-5-one (PMP) have been used for saccharide derivatization [1,2,3,4,5].

Reductive amination is a popular method for introduction of charged/chromogenic/fluorescent labels at the reducing ends of aldoses [4,6]. Reductive amination of the ketone group is less effective than that of an aldehyde, and may generate a new stereocenter to cause complications in separation and structural analysis. Alternatively, we reported previously an efficient method for labeling aldoses with 2,3-naphthalenediamine via the iodine-promoted oxidative condensation reaction to form highly fluorescent naphthimidazole (NAIM) derivatives [7]. Under the similar reaction conditions, 2,3-naphthalenediamine is also applicable to label saccharides of α-ketoacid type, e.g., sialic acid (*N*-acetylneuraminic acid, Neu5Ac), to give benzo [*g*] quinoxaline (BQX) derivatives [8]. It was found that the NAIM-labeled saccharides showed high ionization efficiency in MALDI–TOF MS analysis [9,10]. Several NAIMs derived from pentoses and hexoses are resolved by HPLC on a reverse-phase column [11]. Under optimized conditions, we have also carried out a simultaneous separation of 11 monosaccharide-NAIMs by capillary electrophoresis (CE) using borate buffer [8].

Enantiomers may exhibit different biological activities, even though they exhibit the same physical properties except for having opposite optical rotations. Cyclodextrins (CDs) have been widely used as chiral stationary phase or in ligand-exchange CE for enantioseparation [12,13]. CD and its derivatives, such as sulfated-α-CD, hydroxypropyl-β-CD, heptakis(2,6-dimethyl)-β-CD and heptakis(2,3,6-trimethyl)-β-CD, provide suitable cavities to incorporate the hydrophobic moiety of analytes for resolution of enantiomers by CE in appropriate buffer systems [14]. We have previously reported that the D-/L-enantiomeric pairs of common monosaccharides, such as xylose (Xyl), ribose (Rib), rhamnose (Rha), arabinose (Ara), fucose (Fuc), glucose (Glc), mannose (Man), galactose (Gal), *N*-acetylgalactosamine (GalN), glucuronic acid (GlcA) and galacturonic acid (GalA) were easily converted to their corresponding D-/L-NAIM derivatives, which were resolved on a uncoated fused-silica capillary using sulfated-α-CD as the chiral selector [8].

Since we have established a simple and efficient capillary electrophoretic method for analyzing monosaccharides via their NAIM (for aldoses) and BXQ (for ketoacid sugars) derivatives, we wished to apply this method to determine the absolute configuration and composition of natural carbohydrates in a simultaneous fashion. In this report, we further demonstrate that the NAIM-labeling method can also be used to examine the specificity of glycoside hydrolases and their digestion products.

## 2. Results and Discussion

### 2.1. Compositional Analysis of Ganoderma lucidum Polysaccharide

*G. lucidum* (also called Reshi in Japan or Ling-zhi in China) has been used as a medicinal mushroom in traditional Chinese medicine for thousand years. The polysaccharide constituents of *G. lucidum* are known to possess immunomodulatory functions [15,16,17]. To examine their sugar composition, plant polysaccharides are generally hydrolyzed by enzymatic or chemical methods to release the monosaccharide components, which are then reduced by sodium borohydride and treated with acetyl anhydride to give the corresponding alditol peracetates for GC–MS analysis [18]. The *G. lucidum* polysaccharide RF3, provided by Wyntek Biotech Co. Ltd., Taiwan, has been determined to contain neutral saccharides Xyl, Glc, Man, Ara, Fuc and Gal by using GC–MS method [19]. However, this method fails to detect the acidic saccharide components (e.g., GlcA) without further derivatization. In contrast, both neutral and acidic saccharides can be converted to the NAIM derivatives in one-pot operation and separated by CE [8]. Thus, the NAIM-labeling method can be successfully applied to CE analysis of the sugar compositions in medicinal herbs.

**Figure 1:**
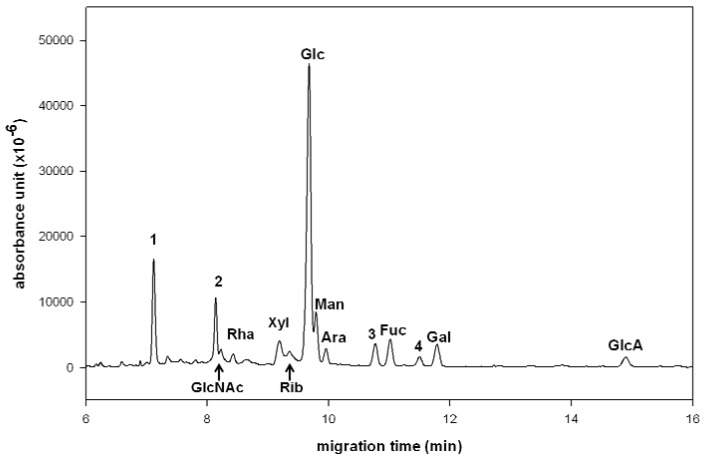
CE analysis of the monosaccharide components (as the NAIM derivatives) in *G. lucidum* polysaccharide. The peaks were identified by comparison with standard monosaccharide–NAIMs. CE conditions: uncoated fused-silica capillary of 30 cm (effective length) × 50 μm id, borate buffer (100 mM, pH 9.0), applied voltage of 12 kV (detector at cathode side), sample loaded by pressure for 3 s, operation temperature at 25 °C, and detection wavelength at 254 nm. In addition to the marked saccharides, peak 1 at 7.2 min is the internal standard (2-naphthol), peak 2 is a non-saccharide fore peak, and peaks 3 and 4 are unidentified components.

In this study, the hydrolytic conditions for degradation of the polysaccharides in *G. lucidum* using trifluoroacetic acid (TFA) at 110 °C for different concentrations (1, 2, 4 and 6 M) and incubation times (1, 2, 4, 6 and 24 h were monitored by high pH anion exchange chromatography-pulsed amperometric detection (HPAEC–PAD). It turned out the best yield (~60% by weight) of total monosaccharide components was obtained by incubation with 4 M TFA for a period of 1–6 h. Thus, *G. lucidum* polysaccharide RF3 (3.0 mg) was hydrolyzed under these conditions and then treated with 2,3-naphthalenediamine to form the monosaccharide-NAIM products. Although the NAIM derivatives are fluorogenic compounds, the UV detection is applied in this study because there is no fluorescence detector coupled with our CE instrument. The factors of running buffer, concentration, pH value and applied voltage were examined to attain the best separation on CE. Under optimized conditions, a simultaneous separation of the monosaccharide-NAIM products was realized by using borate buffer (100 mM, pH 9.0) at 12 kV (Figure 1). The standard samples of each monosaccharide-NAIM were prepared and used to identify the peaks appearing in Figure 1 as that described in our previous report [8]. The CE analysis revealed that the polysaccharide RF3 comprised GlcNAc (2.8%), Rha (2.6%), Xyl (6.6%), Rib (4.6%), Glc (51.8%), Man (8.4%), Ara (3.2%), Fuc (5.5%), Gal (4.8%) and GlcA (2.9%), in addition to two unidentified components (~6.8%) appearing at 10.7 and 11.5 min. The carbohydrate composition determined by CE analysis of the NAIM derivatives was similar to that determined by GC–MS analysis of the trimethylsilylated monosaccharide derivatives [19], with finding of new components of ribose, arabinose and glucuronic acid in this study.

It is known that a diol can react with boronic acid to form a diol–borate complex [20]. Using borate buffer, the migration sequence of monosaccharides appeared to be affected primarily by the extent of formation of the five-membered diol–borate complex [21]. The monosaccharide possessing *cis*-oriented vicinal diol moiety favored the formation of the diol–borate complex over the isomer with a *trans*-oriented diol. Interestingly, the monosaccharide-NAIMs also exhibited a migration trend similar to their parent monosaccharides, presumably due to the rigid skeleton of saccharide-NAIMs as shown by an X-ray diffraction analysis [7]. Thus, Glc-NAIM devoid of *cis*-oriented vicinal diol moiety migrated fastest on CE, followed by Man-NAIM containing 2,3-*cis*-(OH)_2_, Ara-NAIM, Fuc-NAIM and Gal-NAIM containing 3,4-*cis*-(OH)_2_. GlcA-NAIM containing a carboxylic group had the longest migration time on CE.

### 2.2. Compositional Analysis of Dendrobium huoshanense Polysaccharide

Due to a lack of electric charge and chromophores, separation of enantiomeric saccharides by chromatography is problematic without chemical derivatization. The enantioseparation of fungi/plant metabolites by CE has been established [22], but CE determination of the absolute configuration of plant saccharides is less mentioned. We demonstrate herein that the absolute configuration and composition of saccharides can be determined simultaneously by CE analysis via the NAIM derivatives. This method was successfully applied to investigate the polysaccharides extracted from *D. huoshanense*, which is a tonic medicinal herb with immunomodulatory functions to stimulate some growth factors *in vitro* [23]. Thus, the mucilage polysaccharide of *D. huoshanense* (3.0 mg) was hydrolyzed with 4 M TFA to liberate the monosaccharide components (~65% yield), which were then converted to the corresponding NAIM derivatives. 

**Figure 2:**
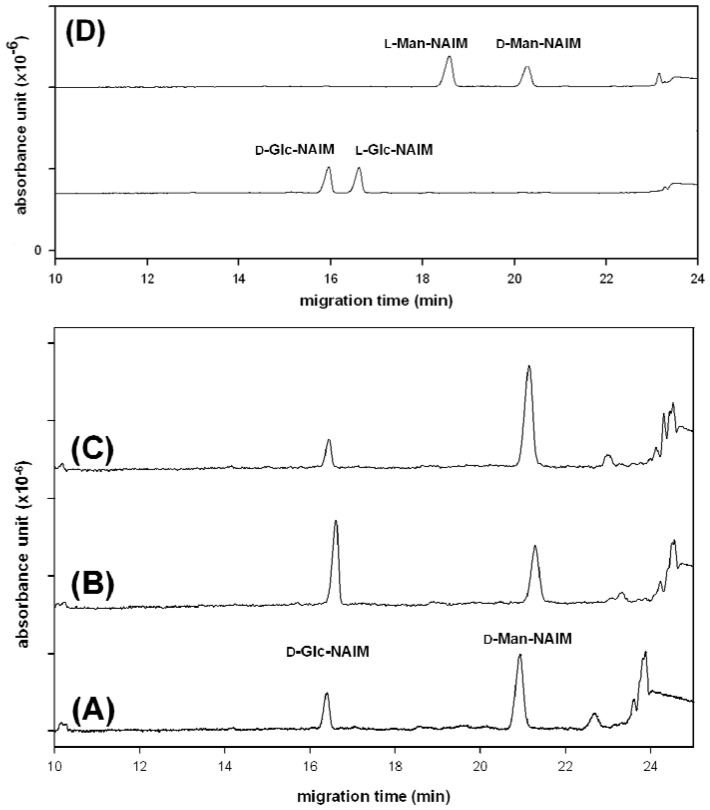
CE analysis of the monosaccharide components (as the NAIM derivatives) in *D. huoshanense* (DH) polysaccharide. (**A**) the hydrolysate of DH polysaccharide with NAIM tag; (**B**) the hydrolysate with D-Glc-NAIM spiked; (**C**) the hydrolysate with D-Man-NAIM spiked; and (**D**) resolution of enantiomeric pairs of Glc-NAIM and Man-NAIM. The NAIM derivatives of incomplete hydrolytic oligosaccharides appeared after 22 min. CE conditions: phosphate buffer (300 mM, pH 3.0), sulfated-α-CD (10 mg/mL) as the chiral selector, applied voltage of 15 kV (detector at cathode side), uncoated fused-silica capillary of 30 cm (effective length) × 50 μm id, sample (0.2 µmol) loaded by pressure for 5 s, operation temperature at 30 °C, and detection wavelength at 254 nm.

Although borate buffer is a good electrolyte for the composition analysis of monosaccharide-NAIMs by CE (Figure 1), the enantiomers of monosaccharide-NAIMs could not be resolved well in borate buffer using sulfated-α-CD, hydroxypropyl-β-CD, heptakis(2,6-dimethyl)-β-CD or heptakis(2,3,6-trimethyl)-β-CD as the chiral selector. We found that using phosphate buffer (pH 3.0), instead of borate buffer, the enantiomers of monosaccharide-NAIMs were resolved on CE (Figure 2D) by formation of inclusion complexes with sulfated α-CD [8]. 

It seemed that the electrophoretic pattern of monosaccharide-NAIMs depended on the ionic strength of the phosphate buffer, which affected the running current during the electrophoresis. The enantioseparation in CE strongly depended on the stability of the diastereomeric ternary complex [24]. Thus, phosphate acted as a central ion to bind the hydroxyl groups in the monosaccharide; in the meantime, cyclodextrin provided a suitable cavity to incorporate the hydrophobic moiety of NAIM. At high ionic concentration, the analyte enantiomers would have longer migration time to interact with phosphate and sulfated-α-CD to give higher resolution. Therefore, high concentrations of phosphate buffer and sulfated-α-CD were needed to obtain a good resolution of enantiomers [25]. However, the migration time of analyte also increased when the concentrations of buffer were increased. To make a compromise, the mixture containing monosaccharide-NAIMs derived from the lysate of *D. huoshanense* polysaccharide was subjected to CE analysis in 300 mM of phosphate buffer (pH 3.0) using 10 mg/mL of sulfated-α-CD as the chiral selector. Under such conditions (at 30 °C with an applied voltage of 15 kV), D-Man-NAIM (73.2%) and D-Glc-NAIM (26.8%) were identified unambiguously by CE analysis (Figure 2A–C). This result of sugar composition in *D. huoshanense* polysaccharide was consistent with that determined by GC–MS and HPAEC–PAD methods [23]. However, the GC–MS and HPAEC–PAD analyses do not provide the information for the absolute configuration of saccharides. In comparison, our current method has advantages in simple chemical modification of monosaccharides to their NAIM derivatives for rapid CE analysis to determine the composition and absolute configuration in one shot.

### 2.3. CE Analysis for Enzymatic Digestion of Oligosaccharides

**Figure 3:**
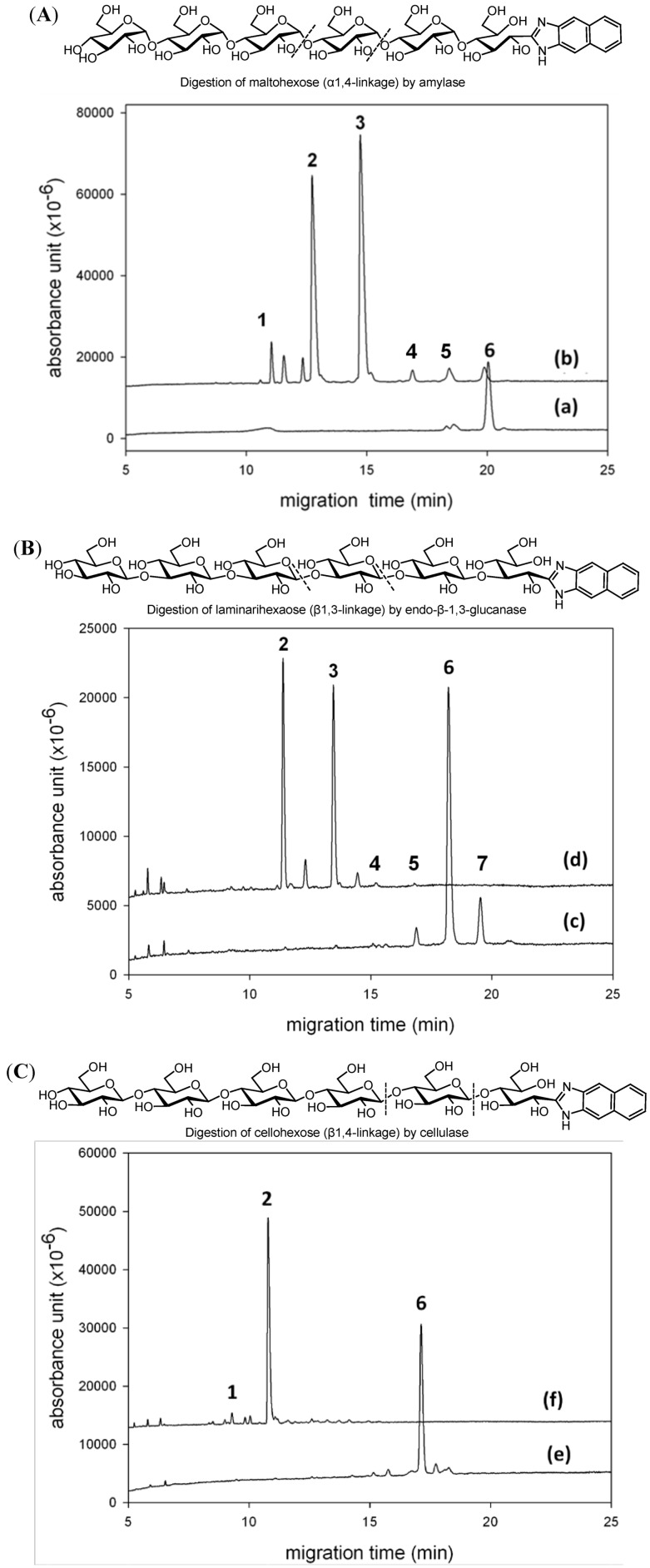
CE analysis of enzymatic digestion of oligosaccharides. The peaks were identified by comparison with standard saccharide–NAIMs. (**A**) Electropherograms of maltohexaose-NAIM derivative (trace a) and after digestion by α-amylase (trace b). Peaks: 1 = glucose-NAIM; 2 = maltose-NAIM; 3 = maltotriose-NAIM; 6 = maltohexaose-NAIM. The peaks at 11.6 and 12.4 min are not identified. (**B**) Electropherograms of laminarihexaose-NAIM derivative (trace c) containing minor components of pentamer and heptamer, and after digestion by endo-β-1,3-glucanase (trace d). Peaks: 2 = laminaribiose-NAIM; 3 = laminaritriose-NAIM; 4 = laminaritetraose-NAIM; 5 = laminaripentaose-NAIM; 6 = laminarihexaose-NAIM; 7 = laminariheptaose-NAIM. The peaks at 12.2 and 14.3 min are not identified. (**C**) Electropherograms of cellohexaose-NAIM derivative (trace e) and after cellulase digestion (trace f). Peaks: 1 = glucose-NAIM; 2 = cellobiose-NAIM; 6 = cellohexaose-NAIM. CE conditions: uncoated fused-silica capillary of 30 cm (effective length) × 50 μm id; phosphate buffer (300 mM, pH 3.0); applied voltage of 15 kV (detector at cathode side); sample loaded by pressure for 5 s; detection wavelength at 254 nm.

A glycoside hydrolase would specifically digest its oligosaccharide substrate, and the CE analysis of the chromogen/fluorescence-labeling oligosaccharide would provide useful structural information of the enzymatic reaction. We chose three glucohexaoses with different glycosidic linkages for NAIM derivatization to further demonstrate the application of NAIM labeling in study of enzymatic digestion (Figure 3). Maltohexose-NAIM having α-1,4-linkages was digested by α-amylase to give maltose-NAIM and maltotriose-NAIM as the major digestion products according to the CE analysis (Figure 3A). This result was consistent with the action of α-amylase to break down amylose into maltotriose and maltose as the ultimate products. The CE analysis indicated that maltohexose-NAIM was inert to endo-β-1,3-glucanase (laminarinase) or cellulase (data not shown). Laminarihexaose-NAIM having β-1,3-linkages was digested by endo-β-1,3-glucanase to give laminaridiose-NAIM and laminaritriose-NAIM (Figure 3B), whereas cellohexose-NAIM having β-1,4-linkages was digested by cellulase to give predominantly cellobiose-NAIM accompanied by a small amount of glucose-NAIM (Figure 3C). Our present method is potentially useful to monitor the activity of various glycanases and to determine the structures of oligo- and polysaccharides released from glycoproteins and glycolipids. Glucan-NAIMs can be the proper substrates for screening various glucanases, and the CE analysis can serve to monitor the degradation products with good sensitivity.

### 2.4. CE Analysis of Heparin Disaccharides with NAIM Labeling

Heparins are important pharmacological medicines used as blood anticoagulants and antithrombins [26]. Identify of the structural features and purity is essential to the safety of using heparins in medical treatments. CE, particularly in reversed polarity mode, is a useful method for separation of heparins [27,28]. For example, analysis of underivatized heparin disaccharides has been carried out by reverse polarity CE using 15 mM phosphate buffer at pH 3.5 with UV detection at 232 nm [29,30]. Derivatization of the heparin oligosaccharides with fluorescent tags can significantly improve both the detection sensitivity and selectivity. Some heparin oligosaccharides have been derivatized to incorporate fluorescent tags by reductive amination to improve CE detection [31]. However, the structural elucidation of heparins may still be problematic due to the high charges and the possible occurrence of numerous isomers such as alternate disaccharide sequences of GlcA and GlcN residues with further *O*-sulfation, *N*-sulfation, and *N*-acetylation [32].

**Figure 4:**
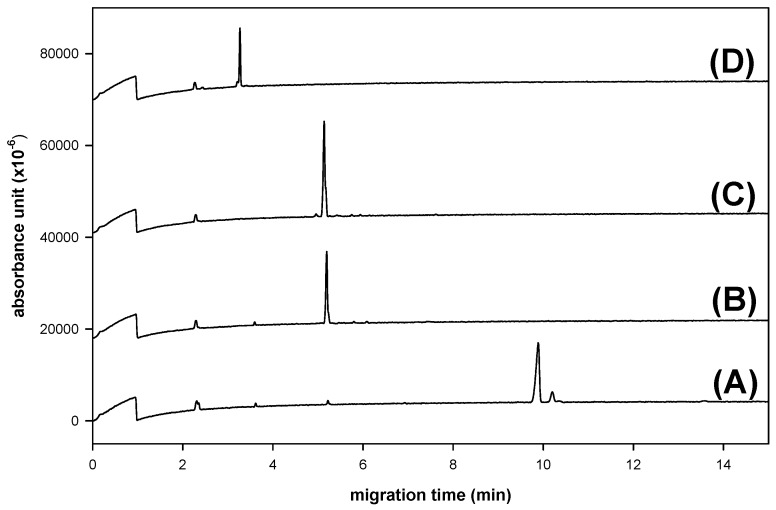
CE analysis of heparin disaccharide-NAIMs: (**A**) α-∆UA-2S-[1→4]-GlcNAc-6S, IA–NAIM); (**B**) α-∆UA-[1→4]-GlcNAc-6S, IIA–NAIM); (**C**) α-∆UA-2S-[1→4]-GlcNAc, IIIA–NAIM; and (**D**) α-∆UA-[1→4]-GlcNAc; IVA–NAIM. CE conditions: uncoated fused-silica capillary of 30 cm (effective length) × 50 μm id, phosphate buffer (300 mM, pH 8.5); applied voltage of 20 kV (detector at cathode side); sample (0.2 µmol) loaded by pressure for 3 s, operation temperature at 25 °C, and detection wavelength at 254 nm.

Reductive amination of saccharides may interfere with other functional groups, such as carboxylic acid, *N*-acetyl and sulfate in heparins. In contrast, saccharides containing carboxylic acid and *N*-acetylgroups (e.g., GlcA and GlcNAc) can be effectively labeled with 2,3-naphthalenediamine [7,8]. Among 12 commercially available heparin disaccharides, four classes are defined: IA contains two sulfate groups at the 2-position of 4-deoxy-L-*threo*-hex-4-eno-pyranosyluronic acid (∆UA) and the 6-position of GlcNAc, IIA contains one sulfate group at GlcNAc 6-position, IIIA contains one sulfate group at ∆UA 2-position, and IVA contains no sulfate group. As a preliminary study toward the structural determination of heparins, we first chose heparin disaccharides IA, IIA, IIIA and IVA for NAIM-labeling to investigate the conditions of CE analysis. The NAIM derivatives were smoothly prepared in high yields by the iodine-promoted condensation with 2,3-naphthalenediamine without interference with sulfate and other functional groups in heparin disaccharides. The NAIM derivatives of heparin disaccharides were then subjected to CE analysis in phosphate buffer (Figure 4). The migration time increased with the number of sulfate groups, *i.e.*, IVA–NAIM (no sulfated group) < IIIA–NAIM (one sulfated group at ∆UA) ~ IIA–NAIM (one sulfated group at GlcNAc) < IA–NAIM (two sulfated groups at ∆UA and GlcNAc). Nonetheless, IIA–NAIM and IIIA–NAIM could not be separated under the CE conditions shown in Figure 4. 

## 3. Experimental

### 3.1. Chemicals and Materials

D-L-Glucose (Glc), D-/L-galactose (Gal), D-/L-mannose (Man), D-/L-arabinose (Ara), D-/L-ribose (Rib), D-/L-xylose (Xyl), D-/L-fucose (Fuc), L-rhamnose (Rha), D-*N*-acetyl glucosamine (GlcNAc), D-glucuronic acid (GlcA), sulfated α-CD, 2-naphthol (used as the internal standard, IS), amylase (*Bacillus licheniformis*), laminarinase (*Trichoderma species*) and cellulase (*Trichoderma vuride*) were purchased from Sigma-Aldrich (St. Louis, MO, USA). Maltohexose, laminarohexose and cellulohexose were purchased from Supelco (Bellefonte, PA, USA). Heparin disaccharides (α-∆UA-2S-[1→4]-GlcNAc-6S; IA), (α-∆UA-[1→4]-GlcNAc-6S; IIA), (α-∆UA-2S-[1→4]-GlcNAc; IIIA), and (α-∆UA-[1→4]-GlcNAc; IVA) were purchased from Dextra Ltd., where ∆UA = 4-deoxy-L-*threo*-hex-4-eno-pyranosyluronic acid; GlcN = D-glucosamine; Ac = acetyl; 2S = 2-sulfate, and 6S = 6-sulfate, respectively. DMSO, 2,3-naphthalenediamine, acetic acid (AcOH) and iodine were purchased from Acros (NJ, USA) and all materials are analytical grade. Disodium tetraborate, sodium dihydrogen phosphate, Tris buffer, hydrochloric acid (HCl) and sodium hydroxide (NaOH) were purchased from Merck (Darmstadt, Germany). Milli-Q water (Millipore, Bedford, MA, USA) was used for the preparation of buffer and the related aqueous solution.

### 3.2. Synthesis of Saccharide–Naphthimidazole Derivatives

The saccharide-NAIM derivatives were synthesized through the Schiff base formation between the 2,3-naphthalenediamine and the aldehyde functionality at the reducing end of aldoses, followed by iodine-catalyzed oxidative condensation of the intermediate imidazoline to the stable NAIM products [7]. Briefly, a solution of aldose in aqueous AcOH was treated with 2,3-naphthalenediamine (1 equiv.) and iodine (10 mol%) at room temperature. The reaction completed in 6–18 h as monitored by TLC or NMR analysis, depending on the various types of aldoses. The mixture was concentrated under reduced pressure, diluted with water, and then triturated with ethyl acetate to give the desired saccharide-NAIM product in high yield (80–96%). The saccharide-NAIM products were practically pure for CE analysis.

### 3.3. Instrument and Experimental Conditions for Capillary Electrophoresis

CE analyses were performed using Beckman Coulter capillary electrophoresis system (model P/ACE MDQ, Fullerton, CA, USA) equipped with a UV detector at 254-nm wavelength. Separation of saccharide-NAIM derivatives was conducted on an uncoated fused-silica capillary [40.2 cm (30 cm effective length) × 50 μm id, Polymicro Technologies, USA]. In a typical CE analysis of saccharide-NAIM derivatives, the BGE was borate buffer (100 mM, pH 9.0). The sample (0.2 µmol) was injected by pressure (0.5 psi for 3 s) at the anodic end of the capillary, and constant voltages of +12 kV and 20 kV were applied during analyses. After CE analysis, each new sample running was conditioned with 0.1 M NaOH for 3 min, water for 3 min and running buffer for another 5 min. For separation of the enantiomers of saccharide-NAIM derivatives, the CE analyses were performed in the BGE containing phosphate buffer (300 mM, pH 3.0) and sulfated α-CD (10 mg/mL) as the chiral selector at a constant voltage of +15 kV.

### 3.4. Hydrolysis of Polysaccharides from Medicinal Herbs

To obtain the component monosaccharides from polysaccharides of herbs, the commercial products of *G. lucidum* polysaccharide (RF3, Wyntek, Taipei, Taiwan) and *D. huoshanense* polysaccharide (YFY Biotech, Taipei, Taiwan) were pretreated with 4 M TFA at 110 °C for 6 h. The hydrolysate was collected, and concentrated under reduced pressure. The crude hydrolysate was labeled with 2,3-naphthalenediamine by the above-described procedure to give the saccharide-NAIM derivatives for CE analysis. 

### 3.5. Validation of Experimental Data

The quantitative determination of saccharide-NAIM derivatives was validated. Calibration curves were obtained by the corrected peak area ratio of each saccharide-NAIM derivative to internal standard (IS) as ordinate (*y*) *versus* the concentration of each saccharide-NAIM derivative as abscissa (*x*). The limit of detection (LOD) was assessed precisely. The precision and accuracy of the intra- (n = 3) and inter-day (n = 5) analyses were evaluated by analyzing three different concentrations of each saccharide-NAIM derivative and five different concentrations of each saccharide-NAIM using 2-naphthol as IS. The *r^2^* values (>0.9969) of the regression curves indicated high linearity in intra- and inter-day analysis. The RSD and relative error (RE) were calculated to estimate the precision and accuracy of the proposed method by analyzing three different concentrations. The RSD and RE values in intra- and inter-day analysis were less than 8.2% and 9.1%, respectively. The results represented good reproducibility and reliability. 

## 4. Conclusions

We have demonstrated that labeling of monosaccharides as the corresponding NAIM derivatives provides an expedient way for composition analysis and enantioseparation on capillary electrophoresis. Transformation of various neutral and acidic saccharides, including those containing carboxyl, acetamido and sulfate groups, to their NAIMs is easily performed in an environmentally-friendly manner. The high-yielding product is stable over a broad pH range and suitable for CE analysis without further purification. Even less than 0.2 μmol of saccharide is readily subjected to derivatization with 2,3-naphthalenediamine and detection by CE equipped with a UV detector in our current laboratory setup. As the NAIM derivatives of saccharides are fluorescent, the LOD can be lowered providing a fluorescence detector is available to couple with CE.

This NAIM labeling method is applicable to determine the composition of saccharides in medicinal herbs with good linearity and short analysis time. Furthermore, CE using sulfated-α-CD as the chiral selector allows enantioseparation of saccharide–NAIMs for a simultaneous determination of sugar composition and absolute configuration. Together with the specific enzymatic reactions of various glycoside hydrolases on the NAIM derivatives of oligo- and polysaccharides, CE analysis of the digestion products provides plenty information to deduce the structures of the parent saccharides. Alternatively, our current method can also be utilized to monitor the type and activity of various glycanases.

Though heparin dissachrides could be successfully derived with the NAIM-labeling method, this method has a limitation because we were unable to find suitable CE conditions for separation of IIA–NAIM and IIIA–NAIM with the same degree of sulfation.
